# Alcohol and energy drinks: a pilot study exploring patterns of consumption, social contexts, benefits and harms

**DOI:** 10.1186/1756-0500-5-369

**Published:** 2012-07-23

**Authors:** Amy Pennay, Dan I Lubman

**Affiliations:** 1 Research Fellow, Centre for Alcohol Policy Research, Turning Point Alcohol and Drug Centre, Eastern Health; Adjunct Lecturer, Eastern Health Clinical School, Monash University, Melbourne, Australia; 2 Director, Turning Point Alcohol and Drug Centre, Eastern Health; Professor, Addiction Studies and Services, Monash University, Melbourne, Australia

**Keywords:** Alcohol, Energy drinks, Stimulant, Policy, Australia

## Abstract

**Background:**

Young people around the world are increasingly combining alcohol with energy drinks (AEDs). However, as yet, limited research has been conducted examining this issue, particularly in terms of exploring patterns of consumption, social practices and the cultural contexts of AED consumption. We sought to understand how AEDs are used and socially constructed among young people.

**Methods:**

We conducted 25 hours of observation in a variety of pubs, bars and nightclubs, as well as in-depth interviews with ten young people who regularly consumed AEDs during a session of alcohol use.

**Results:**

In this pilot study, participants were highly organised in their AED consumption practices and reported rarely altering this routine. Some young people consumed upwards of eight AEDs on a typical night, and others limited their use to between three and five AEDs to avoid unpleasant consequences, such as sleep disturbances, severe hangovers, heart palpitations and agitation. Wakefulness and increased energy were identified as the primary benefits of AEDs, with taste, reduced and increased intoxication, and sociability reported as additional benefits. Young AED users were brand sensitive and responded strongly to Red Bull imagery, as well as discounted AEDs. Finally, some young people reported substituting illicit stimulants with energy drinks.

**Conclusions:**

Combining energy drinks with alcohol is now a normalised phenomenon and an integral and ingrained feature of the night-time economy. Despite this, many young people are unaware of recommended daily limits or related harms. While some young people consume AEDs to feel less drunk (consistent with motivations for combining alcohol with illicit stimulants), others report using AEDs to facilitate intoxication. While preliminary, our findings have relevance for potential policy and regulatory approaches, as well as directions for future research.

## Background

There is a growing body of evidence, both in Australia and internationally, demonstrating increasing consumption of alcohol in combination with energy drinks (AEDs) among youth populations [1]. Consuming alcohol with stimulants has long been an issue for public health given the potential for increased harms [2,3]; however, given the relative recency of combining alcohol with energy drinks, limited research has been conducted examining its benefits and harms in a wider frame.

Energy drinks are caffeinated beverages designed to provide a boost of energy or enhance alertness [4,5]. Energy drinks first appeared in Europe and Asia in the 1960s but did not become popular until the most widely known brand, Red Bull, was released in Austria in 1987; hitting the US market in 1997. By 2006, there were over 500 brands of energy drinks around the world, with sales exceeding $500 million per annum in the US [6]. Around the early 2000s, energy drinks became a popular mixer with alcohol, particularly with spirits such as vodka and Jagermeister, and in 2003 pre-packaged or ‘ready-to-drink’ alcohol energy drinks were introduced [7,8].

To date, only three studies, conducted in the US, Canada and Italy, have examined rates of AED use, finding that between one quarter [5,9] and one half of university students reported consuming an AED in the past month [10]. While prevalence rates of AED use in Australia are unknown, 70% of regular ecstasy users (mean age of 24 years), surveyed as part of the Ecstasy and Related Drugs Reporting System, had consumed an AED in the past twelve months, with two thirds of this latter group reporting that they consumed AEDs weekly or monthly. This sample also reported consuming three AEDs in their last session of alcohol and/or drug use, exceeding the recommended intake of two energy drinks per day [11].

There is some evidence to suggest that combining energy drinks with alcohol leads to increased alcohol consumption. US and Canadian studies have demonstrated that students who had consumed an AED in the past month reported more than twice as many heavy episodic drinking days and drank significantly more during a typical session than those who consumed alcohol alone [5,9,12]. A portal study, in which young people were interviewed and breathalysed leaving licensed venues between 10 pm and 3 am, found that participants who had consumed AEDs were 3.3 times more likely to have a blood alcohol concentration of 0.08 or more compared with those drinking alcohol alone. Those consuming AEDs were also more likely to exit the venue later in the evening, drink for a longer period of time and consume more drinks [13].

Of the existing survey studies that have been conducted on AEDs, only one has explored motivations for use, with 55% of university students reporting mixing energy drinks with alcohol to hide the flavour of alcohol, while 15% reported consuming AEDs in order to be able to drink more and feel less drunk [5]. In relation to harms, the web-based surveys conducted in the US and Canada found that in the past twelve months, those students who consumed AEDs (as opposed to alcohol without energy drinks) were at increased risk of (i) passing out from drinking or drug use, (ii) taking advantage of someone else sexually or being taken advantage of, (iii) having unprotected sex with someone not well known to them, (iv) riding home with a driver who has been drinking, (v) being in a verbal fight and (vi) being injured or hurt [5,9].

To date, only one qualitative study has been conducted investigating young people’s perceptions and experiences of AED use, and this is the only published Australian study on AEDs [8]. Twenty one university students, aged 18–25 years, were interviewed in focus groups. The majority reported drinking AEDs for a number of perceived benefits, including energy, sociability and taste. While the students also reported some negative consequences associated with AED use, such a difficulty sleeping, worse hangovers and aggression, the benefits of AEDs were perceived to outweigh the harms.

Aside from a handful of university-based studies [5,8-10], limited research is available regarding the social and cultural contexts of AED consumption, and in what ways, amounts, patterns, frequencies and locations they are being used. In addition, no information exists around the serving practices and marketing of these products, their social and cultural accommodation and the potential policy and regulatory responses that might be developed to respond to this issue. Undertaking sessions of observation and conducting in-depth interviews, two methods which have not yet been utilised among AED users, we sought to describe how AEDs are used and represented in everyday life and practice, as well as examining the social meanings and practices that underpin their use [14,15]. Previous studies utilising observational methods have provided culturally relevant policy implications [16-20]. Other population-based data collection approaches, such as epidemiology, are not able to investigate the meanings that individuals ascribe to various social practices [21-23]. Only by developing a deeper understanding of the meanings, contexts and practices of AED use can appropriately structured interventions be created and delivered. In this paper, we present findings from a pilot study, involving sessions of observation and in-depth interviews, that builds on our understanding of the social and cultural contexts of AED use in Australia, and points to implications for policy and future research.

## Methods

Ethical approval to conduct the study was obtained from the Eastern Health Research and Ethics Committee (E46/1011). Data collection occurred over a six-month period in 2011 (January to June), and involved two components: sessions of observation and in-depth interviews.

### Sessions of observation

Sessions of observation [24-26] were conducted by the first author (AP) in a variety of pubs, bars and nightclubs in Melbourne, Australia. Sessions of observation took place on five separate occasions between the hours of 9 pm and 4 am, with each session lasting a minimum of five hours. The observation component of the research was exploratory in nature, with free text fieldnotes being completed throughout the night. Particular attention was paid to AED consumption practices including amounts, combinations and frequencies; particular drinking practices (i.e., ‘shots’, ‘rounds’ ‘chugging’); specific behaviours, such as dancing, talking, humour, annoyance and aggression; the marketing of AEDs; serving practices of staff; and any noticeable benefits and harms of consumption (ascertained through observation and informal conversations with patrons).

Observations involved interaction with patrons where possible. This involved the researcher casually interacting with drinkers; for example, by being friendly at the bar with patrons who were ordering AEDs and enquiring about the contents and motivations for use (but not informing the patron they were conducting research). In each venue, particular groups of people were selected for detailed observation of consumption and other behavioural habits, but the general behaviour of all patrons were noted where possible. Sessions of observation were as unobtrusive as possible to maximise the naturalistic setting. Detailed fieldnotes were taken during and immediately following sessions of observation [27,28]. When notes were taken during a session of observation, they were done so in a concealed way (i.e., on an iPhone) so as to ensure the setting remained naturalistic and those being observed were unaware of this practice.

### In-depth interviews

To complement data gathered through sessions of observation, in-depth interviews [29,30] were conducted with ten young (aged 21–31 years) consumers of AEDs who regularly consumed at least two AEDs during a session of alcohol use. We utilised a purposive sampling approach and targeted people between the ages of 18–35 given that the limited epidemiological research available shows that AED consumers are in this age bracket [5,9,10]. Interviewees included five participants who were given an information card by the researcher at the completion of sessions of observation, and five other consumers of AEDs who were accessed through a process of email snowballing. Patrons in venues who were consuming AEDs were given a card with details about the study and invited to contact the researcher if they wished to participate in an interview. In addition, an email was sent out to personal contacts of the first author with a direction to forward the email on to broader personal networks. The email invited eligible potential participants (over the age of 18, regular consumers of AEDs) to contact the researcher if they were interested in participating in an in-depth interview.

All participants signed a written information and consent form prior to commencing the interview. In-depth interviews were conducted in a private space convenient for both the interviewer and interviewee. Interviews lasted between 30 and 60 minutes and participants were reimbursed AU$30 to compensate for time and travel costs. The interview schedule was semi-structured, enabling a certain level of control over the questions, while also allowing responses to dictate the flow of conversation and issues arising [31]. The interview schedule was informed by a review of the literature on AEDs and covered demographics; patterns of alcohol use; patterns of energy drink use; patterns of AED use (frequency and amount); age of first use of these drinks; duration of use; locations of use; preference of beverages; a detailed description of the last session of AED use; motivations for AED use; perceived interactional effects of AEDs; social, cultural and economic influences on AED use; use of pre-packaged AEDs; common harms experienced from AEDs including acute and next-day harms; impact of AED use on lifestyle and daily functioning; perceptions of risky behaviours associated with AED use, and intended future use of AEDs. When participants noted particular benefits or negative consequences from AED use, the researcher followed up with a question asking how this differed from occasions when only alcohol was consumed.

### Sample characteristics

Six of the interviewees were male and the mean age of participants was 25 (range 19–31). Interviewees either worked (n = 8) or studied (n = 2) full time, and owned their own home (n = 1), lived in a rental property (n = 7) or lived with their parents (n = 2). Participants might be considered ‘socially included individuals’ in the sense that they were well-integrated young people with ongoing ties to mainstream society through work and study [32,33].

### Analysis

Data collected via observation and in-depth interviews were stored and analysed using NVivo9, a qualitative software package that enables thematic and content analysis of large amounts of text [34]. Our approach to data collection and analysis was inductive [35,36], with no preconceived notions held about what the findings of the research would be. Combined content and thematic analyses were deemed the most suitable way of systematically analysing key themes across the two data sources. First, a content analysis was performed which involved establishing a list of categories or themes that were commonly identified across the fieldnotes and interviews, and then counting the number of times that these themes were evident [37]. Six primary categories were identified in the content analysis. Following this, we sought to explore in more depth the importance and meaning of each of the categories through a process of thematic analysis [38].

Six key themes were identified in the content and thematic analysis: (i) patterns of consumption, (ii) normalisation of AED, (iii) marketing and promotions, (iv) motivations for AED use, (v) energy drinks as substitution for illicit stimulants and (vi) negative consequences of AED consumption.

## Results

### Patterns of consumption

For the purpose of analysis, one AED corresponds to one ‘standard drink’ of alcohol (10 g of ethanol) mixed with half a can of energy drink (125 mL, which is approximately 40 g of caffeine) [1].

During interviews, AED consumers were asked to describe their patterns of AED consumption on a typical weekend session of alcohol use. Interestingly, participants were highly organised in their AED consumption practices and reported rarely altering this routine. This pattern had become a learned experience, one that they had established after a trial and error phase early in their use of AEDs. This learned routine was based on achieving maximum benefits from AEDs and avoiding or minimising negative consequences associated with their use.

The most commonly reported AED consumption practice (reported by seven of the ten participants) involved the consumption of between two and five AEDs over the course of the night. The evening typically began with one or two AEDs as an initial “booster”, followed by a period of non-AED alcohol use (such as beer, wine or spirit consumption). AEDs were consumed again, later in the evening (around midnight), when they began to feel tired or were drawn to a beverage with a sweeter taste. This later session usually involved drinking between two and five AEDs; the most popular combinations included “bombs” such as Jagerbombs (Jagermeister and energy drinks) and Skittlebombs (Cointreau and energy drinks), or mixers of vodka and energy drinks. For example:

“I’ll often have one [Jagerbomb] before I go out or when I first get to the pub, and then I’ll have maybe five or six beers before I start drinking spirits, such as bourbon and coke or vodka and some type of drink and mixed drinks, and then usually have one [vodka with energy drink] when I start to get tired, around midnight, or I start to get sick of other drinks. Because I like the taste of it so much, I’ll have it when I start to get sick of other things, but also when I start to get bored with the night or, or get tired. I find it gives me a pick-me-up and I can, I feel much more energetic and have more fun again” (Male 28 years).

All seven of these participants noted that they restricted themselves to between two and five AEDs a night so that they would not have trouble sleeping or would not feel too unwell the following day.

There was some exception to this pattern as reported during participant interviews. Three participants reported drinking AEDs constantly throughout their session of alcohol use. One person reported consuming a Jagerbomb every time he went to the bar for a beer, and the final two participants were illicit drug users (methamphetamine, cocaine and/or ecstasy) and both reported mixing illicit stimulants with AEDs (either vodka and energy drinks or Skittlebombs). All three of these participants reported consuming between 8–12 AEDs on a typical night out. For example:

“Yeah pretty much every time we go out we have Jagerbombs and just to start the night off or whatever […] Usually just start off drinking beer to start the night and then probably move on to like a bourbon or a scotch or something like that. Maybe have a couple of lines of speed just before going out and then as we get out I probably get onto Red Bull and vodka, that’s pretty easy to drink and you can pretty much drink them all night and not feel sick” (Male, 29 years).

Both of these patterns of use (drinking between two and five AEDs and drinking eight or more AEDs) were borne out by sessions of observation. For example:

I noticed two groups of people consuming AEDs throughout the night. A group of three women went to the bar twice (once at around 10 pm and then again at around midnight) and ordered Skittlebombs. They all went to the bar together and did the Skittlebombs while ordering other drinks. There was a separate group of men, however, who kept returning to the bar periodically for rounds of Jagerbombs. They seemed to be taking it in shouts. One person would go up to the bar and get a round of Jagerbombs and other drinks (beer and bourbon mixers) and then call his mates over to the bar to do the Jagerbombs. After half an hour or so another male from the same group would go up to the bar and they would do the same. They seemed to be racing each other to see who would finish first and the last person to finish would receive some jeering (Fieldnote, April).

### Normalisation of AED

One of the main themes that arose from interviews and sessions of observation was that consuming AEDs is now a ‘normalised’ phenomenon. When asked how many of their friends consumed AEDs, interviewees reported between 50 to 100%. There were no venues attended during sessions of observation that did not sell AEDs. Interviewees confirmed this observation, noting that it is now possible to purchase AEDs in all licensed venues – whereas a number of years ago some venues did not sell energy drinks. It was suggested by one participant, that although she had been drinking AEDs for “nearly ten years”, she had only noticed the drink had become normalised in the past two or three years:

“It has become far more popular to use alcohol and energy drinks combined, definitely the last 18 months […] There seems to be more of energy drinks available and […] they are now essentially standard fare in most clubs. You go to some of the big clubs and the fridge is just essentially all energy drinks, the only thing you can see is energy drinks” (Female, 29 years).

Interestingly, while it was expected that energy drinks would be more popular within certain types of licensed venue environments, such as nightclubs, participants noted that they enjoyed drinking AEDs at home (some always kept the fridge stocked with a six pack of energy drinks and a bottle of Cointreau or Jagermeister so they could have ‘bombs’ before they went out), at suburban pubs, and also in city bars and clubs. This finding was supported by sessions of observation, in which AEDs were as popular in pubs as they were in nightclubs.

### Marketing and promotions

It was commonly regarded by participants that energy drinks and AEDs are marketed cleverly. All participants noted that energy drinks and AEDs are associated with fun and energy. In particular, the link to extreme sports was regularly reported. All ten participants reported that they were not aware of the recommendation that only two energy drinks should be consumed daily and had not seen any public messages discussing the potential harms of energy drinks or AEDs. Three participants said they would welcome more information about the potential harms of energy drinks and their co-consumption with alcohol.

The young people interviewed did not readily reflect on the advertising of energy drinks in combination with alcohol. More commonly, they reflected on brand identification, and even brand saturation. Red Bull was the brand of energy drink that was most commonly observed during sessions of observation. Red Bull branding was evident in almost all licensed venues, from posters, to bar towelling, to ice buckets and refrigerators. When asked about the marketing of energy drinks, all ten consumers reported that they noticed Red Bull imagery in most of the licensed venues they frequented. For example:

“Red Bull’s pretty much everywhere. Like all the fridges would have to have Red Bull in it and Red Bull signs all over it” (Female, 23 years).

Whether this brand saturation is linked to brand preference was not explicitly investigated; however, seven of the ten participants interviewed identified Red Bull as their preferred energy drink (with two saying ‘V’ and one saying ‘Mother’). However, when asked which energy drink they preferred to consume with alcohol, all ten reported Red Bull. When asked their reasons for this, half the sample reported that they had become accustomed to the taste of Red Bull with alcohol given it was the brand most commonly sold in venues, and the other half stated that they believed Red Bull tasted “the best” with alcohol.

The other key element to this was that young people were extremely attracted to promotions on energy drinks and AEDs. While most consumers noted that licensed venues did not regularly offer discounted AEDs, two consumers reported that “Uni bars” and bars within backpacker accommodation were the two types of venues where you could commonly find discounted AEDs, from between $5 and $7 (as opposed to between $11 and $15 in other venues). Others commented that although they often did not see cheap or discounted AEDs, many licensed venues have signs or blackboards listing all the types of ‘bombs’ you can buy, and this often encouraged the impromptu consumption of a Jagerbomb or Skittlebomb, or equivalent. For example:

“If you see a sign then, yeah, you start thinking about it harder […] If you’re in the bar and you see a sign you kind of know you’re going to do one later” (Male, 24 years).

### Motivations for AED consumption

Consumers of AEDs were asked to identify their primary motivation for consuming AEDs, and five main benefits of the beverage combination were reported: wakefulness and energy; taste; counteracting the drowsy effects of alcohol; facilitating alcohol intoxication, and social bonding.

All ten consumers interviewed noted that wakefulness and increased energy was the primary benefit of consuming AEDs. For example:

“I’ve found on a Friday you work so you’re already tired, so you want something to pick yourself up […] that’s one of the reasons why I like to have them on Friday because you are half asleep” (Male, 24 years).

“I’m getting older, so I definitely want to try and keep up with the crowd, like you can’t stay out as long as you used to, so I think it’s just an easy option” (Female, 29 years).

Seven interviewees commented that one of the reasons they consumed AEDs was because they enjoyed the taste of them. It was particularly common for participants to report switching to vodka AEDs after a period of drinking beer or wine for a sweeter, more palatable taste. Participants who wanted to increase their alcohol intoxication by consuming shots also noted that they selected Jagerbombs because they were more palatable than other shots, such as tequila.

Five participants noted that AEDs were beneficial because the energy drink effectively counteracted the effects of the alcohol. When discussing this, participants drew a distinction between drowsy drunkenness and wakeful drunkenness; for example:

“I’ve just got a lot more energy whilst I’m drunk [when consuming AED]. You’re not slurring or a lethargic drunk; you’re an energetic, happy drunk” (Male, 24 years).

“You’re slightly more energetic and chatty. You get to be less of, like, an inebriated drunk” (Male, 21 years).

While some participants noted that AEDs counteracted the effects of alcohol, four participants suggested that AEDs facilitated intoxication over just drinking alcohol alone. These participants indicated that energy drinks enabled the faster consumption of alcohol. This comment was particularly in relation to ‘Jagerbombs’ and ‘Skittlebombs’ (and other types of ‘bombs’) because these drinks are ‘chugged’ or ‘skolled’, and so consuming these drinks enabled participants to reach intoxication at a faster pace. Participants also noted that when they consumed a ‘bomb’ they also bought a ‘chaser’ (such as a beer), so they were drinking more drinks at a faster pace. The following comment was made by a participant after she was asked what differentiates a night out when drinking AEDs from a night without energy drinks:

“I just get really drunk [when I consume AEDs]. Because I think it like, it makes, like, you excited, and then you drink more as well. I find if I have them, like I start on them, then I drink more of other drinks as well. Does that make sense? So, like, you’re more, like if I just start like, chilled, I’ll just like chill on my drink for the night, but if I start like with something hypo then it’ll just make me skoll the rest of my drinks” (Female, 23 years).

The following fieldnote also highlights this theme:

"I noticed three males who appeared to be playing drinking games with each other. I overheard a conversation between them and a female at the bar, and it appeared they had been drinking all day at the horse races. I noticed at one point they were skolling Jagerbombs, and they all went in a shout for a round of Jagerbombs straight after one another (three shouts in a row). They were particularly rowdy and seemed to be having a good time. Their energy seemed to really pick up after consuming these Jagerbombs and they danced and laughed for a while. Two hours later however, the three men were preparing to leave the bar. I heard one of their female friends (possibly a girlfriend) chastising one of the males for ending their night early because he was too drunk. I heard the men talking about desperately needing to find food before going home (Fieldnote, March). 

The fifth and final benefit reported was the social aspect of consuming AEDs. Participants noted that the consumption of ‘bombs’ facilitated social interaction and humour by virtue of attending the bar together in a group, dropping the shot of liquor into an energy drink and skolling, seeing who could finish first. The following fieldnote supports this theme:

"In the corner of the bar there was a small taped off section reserved for a private function. At midnight the small group were preparing to leave and one male ordered approximately fifteen Jagerbombs. He called everyone in the group over to do the Jagerbomb – it was his shout. He yelled out that nobody was allowed to leave until they’d had one. The group all consumed their Jagerbomb at the same time on the count of ‘three’, and everyone cheered when they had finished (Fieldnote, June)."

One participant commented:

“It’s the interaction with everybody else. I think that’s where you get sucked in the most. I’ve got a friend that loves the shots and then all of a sudden you know, you’re all part, not like a team, but you’re all part of a group” (Female, 29 years).

### Energy drinks as substitution for illicit stimulants

One theme that came up regularly among consumers was that energy drinks were often used in place of illicit stimulants, such as methamphetamine or cocaine. This is not to say that the effects of energy drinks were described as the same as illicit stimulants – because participants pointed out that energy drinks were not associated with the ‘rush’ and ‘buzz’ of illicit stimulants – but rather that energy drinks and illicit stimulants share some properties, such as wakefulness and counteracting the drowsy effects of alcohol. For example, the following observation:

"While at the bar, I spoke with Female A who told me that she’d had a big weekend the one before on the “Red Bulls”. She said that she went out with a big group of people on Saturday night who were smoking “pipes” [of methamphetamine] before they went out and she was “off the speed” at the moment, so had resisted, but managed to stay awake all night drinking energy drinks instead, and had a really good night. Her friend, Female B, overheard us and told me that she used to use cocaine and speed but doesn’t anymore, so energy drinks are her substitute when she goes to bars. She said energy drinks can make her feel “speedy” (Fieldnote, April 2011)."

In addition, three participants commented that they were non-drug users, but that most of their social group consumed illicit stimulants. All three of these participants reported mixing energy drinks with alcohol in an attempt to stay out longer with their drug-using friends. For example:

"Interviewer: “Have you ever used illicit stimulants”?

"Participant: “No, that’s why I sort of drink energy drinks to keep up with people that are”.

"Interviewer: “That are using stimulants”?

"Participant: “Yeah, otherwise I can’t keep up” (Female, 27 years).

### Negative consequences of AED use

Participants were also asked to describe any negative outcomes that they had experienced as a result of consuming AEDs, and there were three main harms that participants reported: sleep disturbance, worse hangovers and increased heart rate.

The main problem associated with AEDs, as noted by seven of the ten participants, was that after consuming AEDs it was common for them to fall asleep when they arrived home from a licensed venue, but would then wake up after a number of hours (between one and six) and not be able to get back to sleep. For example:

“Even if I’ve had a heap of them I fall asleep straight away when I get home, but four or five hours later I wake up and there’s just no getting back to sleep. You just, your body feels like it’s dead and hung over and you don’t want to get out of bed and you don’t want to move, but your mind just won’t let you sleep” (Male, 28 years).

It is interesting to note that no participants observed having trouble sleeping upon first arriving home, but only that they had trouble staying asleep. It may be that heavy alcohol intake promotes sleep induction, but the stimulant effects of caffeine take over as the alcohol is metabolised.

Six of the ten participants commented that they felt more “hungover” and unwell the day following an evening of AED use. For example:

“I don’t usually get hangovers, like I don’t get headachy or feel nauseous or anything, but if I’ve had too many energy drinks I just feel terrible and paranoid and, anxious I think it is, cause I just, I don’t know, it’s something, I don’t know what it does, it just makes you feel… weird” (Male, 19 years).

Two participants suggested this was likely to be associated with dehydration, acknowledging that both caffeine and alcohol are diuretics.

The final commonly noted concern was the experience of “racing heart”, “heart palpitations”, “shakiness” and “twitching” either later in the night, after sleeping for a few hours or during the following day. Five participants reported these effects. For example:

“Ah, oh there was one, one night when me and a mate went out and I think we worked out we’d spent close to $200, $250, on vodka and Red Bulls and Jagerbombs that night. We got home at, we didn’t get home til seven or eight o’clock um… my mate slept in another room and I think I’d only been asleep for an hour and I started having panic attacks and my heart was racing and I just, I couldn’t work it, I just felt like I was going to die. And eventually it wore off but it was, it felt horrible and my mate was the same. We just drank way too many of them. It just, you couldn’t get comfortable, you were just wriggling around, heart racing, mind going a thousand miles an hour” (Male, 24 years).

## Discussion

This pilot study extends the limited international literature on AED use by examining patterns of use, social and cultural contexts, and benefits and negative consequences of AED use among a sample of young consumers using a combination of observational research and in-depth interviews. One of the main themes identified was that combining energy drinks with alcohol is now a normalised phenomenon, and AEDs are now an integral and ingrained feature of the night-time economy, used among young people who are pursuing a range of psychoactive experiences in their leisure time [see also [8,39]. This is consistent with Jones et al.’s qualitative study of university students [8], which concluded that young people choose AEDs “because they enable them to enjoy a more intense psychoactive effect of alcohol for a longer period of time” (pg. 3).

In terms of patterns of consumption, some AED users consume upwards of eight AEDs on a typical night, while others limit their use to between three and five AEDs to avoid unpleasant consequences, such as sleep disturbances, severe hangovers, heart palpitations and agitation. The benefits and harms of AEDs experienced in this sample were similar to those identified by Jones et al. [8], which included alertness, energy, sociability, taste, and increased intoxication. These same benefits were noted by our sample, giving increased weight to the findings of both small-scale studies.

In addition, the negative consequences of AEDs as reported by Jones et al. included difficulty sleeping, worse hangovers than when consuming alcohol without energy drinks, aggression, violence, heart palpitations, blackouts, vomiting and twitching [8]. Aside from violence and blackouts, we identified similar harms associated with AED use, which demonstrates that people in different cities (our study was based in the large metropolitan city of Melbourne and Jones et al.’s study was conducted in the regional town of Wollongong), and with different demographic backgrounds (our sample was older and only two were students), are experiencing similar types of harm from AED use.

Our study showed that wakefulness was considered the primary benefit of AEDs, and difficulty sleeping was the main concern. Interestingly, some AED users consume AEDs to feel less drunk (less drowsy and inebriated) and others use AEDs to facilitate drunkenness (through the faster paced consumption of ‘bombs’). Those who are using AED to feel less drunk, might be using energy drinks in the same way that some illicit stimulant users combine alcohol and stimulants to feel in more control when they are drinking [9,40]. In addition, while only two illicit stimulant users were interviewed in this study, they both used AEDs in higher amounts than the remainder of the sample, indicating that, perhaps unsurprisingly, illicit stimulant users are likely to have preference for other types of stimulants [9].

While participants reported many negative consequences associated with AED use, the benefits were noted to outweigh the harms at this point in their lives, a time when they are actively pursuing leisure and pleasure during their weekends and are willing to spend a Sunday feeling hungover or unwell. It is expected that as these young people move into more traditional adult roles such as starting a family, their use of AEDs is likely to decline, similar to the way that young illicit stimulant users reduce their use after they cease nightclubbing [14]. However, further research is needed to examine the trajectory of AED use across the life course.

### Implications for policy

While there is insufficient research evidence to indicate that AEDs should be banned at licensed venues or off-premise liquor stores, our findings do raise a number of issues that are relevant to the policy space. Young people in our sample reported that they were unaware about recommendations related to consuming only two energy drinks per day, and requested more information about the actual harms of AED use so they could make informed decisions about their consumption practices. In Australia, there is currently no information contained on energy drinks stating that they should not be mixed with alcohol. One option here would be to legislate that information about safe consumption practices should be mandatory on labels of pre-packaged or ready-to-drink AEDs, alongside existing mandatory information that specifies the number of standard drinks per container and that alcohol should not be consumed when pregnant. In addition, as has been recommended in a number of other countries, including Canada and Ireland, energy drinks could include information on their packaging highlighting that there may be harms associated with mixing energy drinks with alcohol, or that caution should be exercised if mixing with alcohol [7,41].

Given the emphasis that consumers placed on brand identification and responsiveness to discounted AEDs, we recommend restrictions on the promotions and marketing of AEDs and the sale of discounted AEDs. Such restrictions could be built into existing advertising and marketing guidelines across various jurisdictions, which may include bans on the provision of free drinks, restrictions on ‘happy hours’ and discounted drinks to minimise the risk of rapid, excessive or irresponsible consumption of liquor, and bans on the advertising or promotion of liquor that draws an association between drinking and risk taking (for an Australian example of these guidelines, go to http://www.justice.vic.gov.au).

Finally, given the relative lack of awareness around the potential harms of AEDs and desire for more information, a range of awareness raising activities might also be considered. These might include mass media campaigns via television, radio, print or billboards, emphasising the potential harms of AEDs, or perhaps including these messages within already existing alcohol campaigns. Another alternative is to engage in target awareness raising; for example, raising awareness among AED consumers via posters displayed at licensed venues (behind the bar or in bathrooms) emphasising harms such as disturbed sleep and severe hangovers, as well as the recommended maximum daily energy drink intake.

### Limitations

There are a number of limitations associated with this pilot study that should be considered when reviewing the results. Participants were recruited using a convenience sampling approach and the interview component relied on participants’ self-report. Together with the small sample, this means that it is unlikely that the findings can be generalised to all young consumers of AEDs. While only ten participants were recruited, this number has been shown to be sufficient in other qualitative studies [42-45]. However, we cannot be confident that data saturation [44] was achieved in this study. While acknowledging these limitations, it is important to note that the study has a number of important strengths, including novel data collection methods, which have not been previously used with AED consumers, and a focus on the social practices, patterns of consumption and cultural contexts of AED consumption, which have been previously ignored. As such, this study provides an important preliminary snapshot into the way that AEDs are used, socially constructed and positioned within the context of a ‘big night out’.

### Implications for future research

Given the relative novelty of combining alcohol with energy drinks, this exploratory pilot study has highlighted potential future avenues for research. Clearly, further research is required to explore the social and cultural contexts of AED use, patterns of consumption, benefits and harms. The current study should be replicated in larger and more diverse samples, including various subgroups of AED users. In particular, we found that some AED users consume five or less AEDs a night and others consume over 10 AEDs a night. Some young people are also consuming AEDs while concurrently using illicit drugs. Future research should attempt to gather more detailed information across these different consumption patterns and flesh out the harms and benefits for these young people.

The young people in this research had been regular consumers of AEDs for some time and had developed their patterns of consumption after a period of experimentation in the past. Future research should focus on young people who are still in this period of experimentation; particularly people under the age of 18, as these people might be more likely to experience acute harms from the consumption of AEDs (through over-consumption) and be most susceptible to the marketing and advertising of these products.

Further, given that AEDs are commonly consumed in licensed venues, research is needed to understand the role that AEDs plays for venue workers and police; for example, whether the combination increases alcohol-related intoxication and/or related aggression or violence. Finally, given that participants reported harms such as “racing heart”, “heart palpitations”, “shakiness” and “twitching”, research is needed into the implications of AEDs for emergency services personnel (including paramedics and hospital staff), as well as other health workers.

## Conclusion

Limited research has been undertaken exploring patterns of consumption, social practices and the cultural contexts of AED use. We sought to understand how AEDs are used, experienced and perceived by young consumers using novel data collection methods. Combining energy drinks with alcohol is now a normalised phenomenon and an integral and ingrained feature of the night-time economy. Young people are mixing energy drinks with vodka, Jagermeister and Cointreau (and sometimes concurrently with illicit stimulants) in the context of a ‘big night out’. Motivations to use AEDs include increased energy, reduced inebriation, increased intoxication, improved taste and greater sociability. Negative consequences include sleep disturbance, severe hangovers, heart palpitations and agitation. After a trial and error period, some young people learn to minimise these harms by limiting the number of AEDs they consume. However, many people are unaware of recommended daily limits of energy drinks and AEDs, as well as related harms. As such, a range of regulatory options such as adding more information to packaging and implementing guidelines around marketing and promotions should be considered, and awareness raising activities should be employed to ensure young people are making informed decisions about their consumption practices. Future qualitative research is needed to compliment epidemiological and clinical studies, so as to position these findings within real-world contexts.

## Competing interests

The authors declare they have no competing interests.

## Authors’ contributions

AP was involved in the design of the study, the collection and analysis of data and drafting the manuscript. DL was involved in the design of the study and drafting the manuscript. Both authors read and approved the final version of the manuscript.

## References

[B1] Pennay A Lubman DI Miller P Combining Energy Drinks and Alcohol: A Recipe for Trouble? Aust Fam Physician 2011 40 3 104 107 21597509

[B2] Baker A Lee NK Jenner L Models of Intervention and Care for Psychostimulant Users Monograph Series 2004 2 Australian Government Department of Health and Ageing, Canberra

[B3] Pennings J Leccese A de Wolff F Effects of concurrent use of alcohol and cocaine Addiction 2002 97 773 783 10.1046/j.1360-0443.2002.00158.x 12133112

[B4] Miller KE Energy Drinks, Race, and Problem Behaviors Among College Students J Adolesc Heal 2008 43 490 497 10.1016/j.jadohealth.2008.03.003 PMC2575122 18848678

[B5] O’Brien MC McCoy TP Rhodes SD Wagoner A Wolfson M Caffeinated Cocktails: Energy Drink Consumption, High-risk Drinking, and Alcohol-related Consequences among College Students Acad Emerg Med 2008 15 5 453 460 10.1111/j.1553-2712.2008.00085.x 18439201

[B6] Miller KE Wired: Energy Drinks: Jock Identity, Masculine Norms, and Risk Taking J Am Coll Heal 2008 56 5 481 489 10.3200/JACH.56.5.481-490 PMC2562885 18400659

[B7] Reissig CJ Strain EC Griffiths RR Caffeinated Energy Drinks - A Growing Problem Drug Alcohol Depend 2009 99 1–3 1 10 18809264 10.1016/j.drugalcdep.2008.08.001 PMC2735818

[B8] Jones SC Barrie L Berry N Why (not) alcohol energy drinks? A qualitative study with Australian university students Drug Alcohol Revearly online101111/j1465-3362201100319x 10.1111/j.1465-3362.2011.00319.x 21605204

[B9] Haber P Lintzeris N Proude E Lopatko O Guidelines for the Treatment of Alcohol Problems 2009 Sydney: The University of Sydney

[B10] Oteri A Salvo F Caputi AP Calapai G Intake of energy drinks in association with alcohol beverages in a cohort of students of the School of Medicine of the University of Messina Alcohol Clin Exp Res 2007 31 10 1677 1680 10.1111/j.1530-0277.2007.00464.x 17651468

[B11] Sindich N Burns L Australian Trends in Ecstasy and Related Drug Markets 2009: Findings from the Ecstasy and Related Drugs Reporting System (EDRS) Australian Drug Trends Series 2010 Sydney: National Drug and Alcohol Research Centre, University of New South Wales

[B12] Price SR Hilchey CA Darredeau C Fulton HG Barrett SP Energy drink co-administration is associated with increased reported alcohol ingestion Drug Alcohol Rev 2010 29 331 333 10.1111/j.1465-3362.2009.00163.x 20565526

[B13] Thombs DL O’Mara RJ Tsukamoto M Rossheim ME Weiler RM Merves ML Event-level analyses of energy drink consumption and alcohol intoxication in bar patrons Addict Behav 2010 35 325 330 10.1016/j.addbeh.2009.11.004 19954894

[B14] Duff C Johnston J Moore D Goren N Dropping, Connecting, Playing and Partying: Exploring the social and cultural contexts of ecstasy and related drug use in Victoria 2007 Melbourne: Premier’s Drug Prevention Council: Department of Human Services Victoria

[B15] Pearson G Normal Drug Use: Ethnographic Fieldwork Among an Adult Network of Recreational Drug Users in Inner London Subst Use and Misuse 2001 36 1&2 167 200 10.1081/ja-100000234 11305351

[B16] Lewis L Ross M A Select Body: The gay dance party subculture and the HIV/AIDS pandemic 1995 London: Cassell

[B17] Thornton S Club Cultures: Music, Media and Subcultural Capital 1996 Hanover: Wesleyan University Press

[B18] Graham K Bernards S Osgood DW Wells S Bad nights or bad bars? Multi-level analysis of environmental predictors of aggression in late-night large-capacity bars and clubs Addiction 2006 101 11 1569 1580 10.1111/j.1360-0443.2006.01608.x 17034436

[B19] Hommel R Tomsen SJT Public drinking and violence: Not just an alcohol problem J Drug Issues 1992 22 3 679 697

[B20] Tomsen S A Top Night: Social Protest, Masculinity and the Culture of Drinking Violence Br J Criminol 1997 37 1 90 103 10.1093/oxfordjournals.bjc.a014152

[B21] Rumbold G Hamilton M Hamilton M, Kellehear A, Rumbold G Addressing drug problems: the case for harm minimisation Drugs in Australia: A Harm Minimization Approach 1998 Singapore: Oxford University Press

[B22] Hammersley M Atkinson P Ethnography: Principles in Practice 1995 New York: Routledge

[B23] Clatts M Welle D Goldsamt L Lankenau S An ethno-epidemiological model for the study of trends in illicit drug use: reflections on the ’emergence’ of crack injection Int J Drug Pol 2002 13 285 295 10.1016/S0955-3959(02)00123-8

[B24] Graham K Wells S Aggression among young adults in the social context of the bar Addiction Res Theor 2001 9 3 193 219 10.3109/16066350109141750

[B25] Becker HS Geer B Participant Observation and Interviewing: A Comparison Soc Appl Anthropol 1957 16 3 28 32

[B26] Adler P Adler P Denzin NK, Lincoln YS Observational techniques Handbook of Qualitative Research 1994 Thousand Oaks, London

[B27] Chiseri-Strater E Sunstein B FieldWorking: Reading and Writing Research 1997 New Jersey: Blair Press

[B28] Fetterman D Ethnography: Step by Step 1989 Newbury Park: Sage Publications

[B29] Johnson JM Gubrium JF, Holstein JA In-depth interviewing Handbook of Interview Research: Context and Method 2001 Thousand Oaks: Sage Publications

[B30] Seidman I Interviewing as qualitative research: A guide for researchers in education and the social sciences 2006 New York: Teachers College Press

[B31] Coleman L Cater S Changing the culture of young people’s binge drinking: From motivations to practical solutions Drug Educ Prev Pol 2007 14 4 305 307 10.1080/09687630601070878

[B32] Hammersley R Khan F Ditton J Ecstasy and the rise of the chemical generation 2002 London: Routledge

[B33] Harling MR The place and meaning of ’controlled’, illicit substance use in the private lives of a group of individuals J Subst Use 2007 12 1 1 12 10.1080/14659890600737208

[B34] Beekhuyzen J Putting the pieces of the puzzle together: Using Nvivo for a literature review. QualIT 2007 — Qualitative Research: From the Margins to the Mainstream 2007 New Zealand: Wellington

[B35] Miles MB Huberman M Qualitative Data Analysis 1994 2 Thousand Oaks: Sage Publications

[B36] Patton MQ Qualitative research and evaluation methods 2002 3 Thousand Oaks: Sage Publications

[B37] Joffe H Yardley L Marks D, Yardley L Content and Thematic Analysis Research methods for clinical and health psychology 2003 London: Sage

[B38] Braun V Clarke V Using thematic analysis in psychology Qual Res Psychol 2006 3 2 77 101 10.1191/1478088706qp063oa

[B39] Measham F Brain K ‘Binge’ drinking, British alcohol policy and the new culture of intoxication Crime Media Cult Int J 2005 1 3 262 283 10.1177/1741659005057641

[B40] Hunt G Evans K Moloney M Bailey N Combining Different Substances in the Dance Scene: Enhancing Pleasure, Managing Risk and Timing Effects J Drug Issues 2009 39 3 495 521 10.1177/002204260903900303 20686669 PMC2913477

[B41] Finnegan D The health effects of stimulant drinks Nutr Bull 2003 28 147 155 10.1046/j.1467-3010.2003.00345.x

[B42] Olsen A Consuming e: Ecstasy use and contemporary social life Contemp Drug Probl 2009 36 175 191 Spring/Summer

[B43] Gould D Tuffey S Udry E Loehr J Burnout in competitive junior tennis players: II. Qualitative analysis Sport Psychol 1996 10 4 341 366

[B44] Guest G Bunce A Johnson L How many interviews are enough? An experiment with Data Saturation and Variability Field Meth 2006 18 1 59 82 10.1177/1525822X05279903

[B45] Morse JM Determining Sample Size Qual Heal Res 2000 70 1 3 5

